# Ovariotomy by Enucleation

**Published:** 1874-01

**Authors:** 


### Ovariotomy by Enucleation.

Dr. Ludlam thus describes a successful operation by the above method in *U. S. Medical and Surgical Journal:*

"I made the incision, as usual, along the linea alba. At first it was only four inches, but it was afterwards enlarged to five inches. There was but little Hæmorrhage. Anteriorly the adhesions were so intimate and firm that it was only by the escape or the abdominal fluid that the lower end of the incision, and the application of Atlee's test that we were certain that the peritoneal cavity had been opened. The sound was passed beneath the umbilicus, but would not glide over the anterior surface of the tumor at all. A slight separation of the adhesions was attempted on each side of the incision, sufficient to prove that they were very compact and very vascular. This fact was so obvious that all the physicians present expressed themselves as satisfied that the operation must have been abandoned, or that the patient's life would have been put in great peril by completing it after the old method. And this state of things caused me to renew my resolution to test the expedient of enucleation.

At a glance it was evident, however, that the mode of performing this operation as first recommended and practiced by Prof. J. F. Miner, of Buffalo,* was impracticable. The tumor could not be turned out upon the abdomen, and the adhesions were in the way of getting at the pedicle. Therefore, in order to separate the cyst, we could not begin "under the central portion of the pedicle," †but had to content ourselves with first detaching it at a point opposite the abdominal incision.

Now this, as you may suppose, was a very delicate matter. The peritoneal layer being very thin, and the cyst-wall likewise, the greatest care had to be exercised in beginning and in completing their dissection and detachment. A very slight incision was first made, and then the handle of the scalpel was

* The American Journal of the Medical Sciences for October, 1872, p. 391.

† This is not always practicable or necessary. adhesions should be separated as they present themselves. The attachments of the pedicle are to be treated in the same way as those of adjacent parts. It is better however, when possible, to commence at the base, avoiding the vascular portion, and to follow out the strands of the pedicle to their termination. Ed.]

used to carry on the separation until it was sufficiently extended to allow of the fingers being employed in the same way. It was only with extreme care and patience that this part of the operation was performed, for the cyst required to be separated in this manner throughout the whole circumference. Indeed it took Dr. Dorion and myself nearly three-fourths of an hour to accomplish this object. And during all this time we exercised the precaution not to lift or disturb the matrix of the tumor lest we might rupture some delicate adhesions on its posterior surface, and thereby cause a concealed internal hæmorrhage.

He says, in review of this case: I am satisfied that Dr. Miner's operation is an invaluable one. Especially is this true where the nature and the extent of the parietal and visceral adhesions render it unsafe and impracticable to remove an ovarian cyst by the more ordinary method. I do not suppose that this operation is suited to all cases indiscriminately; but, in this particular instance, it is evident that my patient owes her life to it and to the careful after-treatment and nursing which she received."

